# Phosphatidylcholine-Specific Phospholipase C as a Promising Drug Target

**DOI:** 10.3390/molecules28155637

**Published:** 2023-07-25

**Authors:** Chatchakorn Eurtivong, Euphemia Leung, Nabangshu Sharma, Ivanhoe K. H. Leung, Jóhannes Reynisson

**Affiliations:** 1 Department of Pharmaceutical Chemistry, Faculty of Pharmacy, Mahidol University, 447 Si Ayutthaya Road, Ratchathewi, Bangkok 10400, Thailand; 2 Auckland Cancer Society Research Centre, The University of Auckland, Private Bag 92019, Auckland 1142, New Zealand; eleu004@uoa.auckland.ac.nz; 3 School of Chemical Sciences, The University of Auckland, Private Bag 92019, Auckland 1142, New Zealand; nabangshu.sharma@scionresearch.com; 4 Scion (New Zealand Forest Research Institute), Te Papa Tipu Innovation Park, 49 Sala Street, Rotorua 3010, New Zealand; 5 School of Chemistry and Bio21 Molecular Science and Biotechnology Institute, The University of Melbourne, 30 Flemington Rd, Parkville, VIC 3052, Australia; ivanhoe.leung@unimelb.edu.au; 6 School of Pharmacy and Bioengineering, Keele University, Newcastle-under-Lyme ST5 5BG, UK; j.reynisson@keele.ac.uk

**Keywords:** cancer, atherosclerosis, inflammation, inhibitors, drug discovery

## Abstract

Phosphatidylcholine-specific phospholipase C (PC-PLC) is an enzyme that catalyzes the formation of the important secondary messengers phosphocholine and diacylglycerol (DAG) from phosphatidylcholine. Although PC-PLC has been linked to the progression of many pathological conditions, including cancer, atherosclerosis, inflammation and neuronal cell death, studies of PC-PLC on the protein level have been somewhat neglected with relatively scarce data. To date, the human gene expressing PC-PLC has not yet been found, and the only protein structure of PC-PLC that has been solved was from *Bacillus cereus* (PC-PLC*_Bc_*). Nonetheless, there is evidence for PC-PLC activity as a human functional equivalent of its prokaryotic counterpart. Additionally, inhibitors of PC-PLC*_Bc_* have been developed as potential therapeutic agents. The most notable classes include 2-aminohydroxamic acids, xanthates, *N*,*N*′-hydroxyureas, phospholipid analogues, 1,4-oxazepines, pyrido[3,4-*b*]indoles, morpholinobenzoic acids and univalent ions. However, many medicinal chemistry studies lack evidence for their cellular and in vivo effects, which hampers the progression of the inhibitors towards the clinic. This review outlines the pathological implications of PC-PLC and highlights current progress and future challenges in the development of PC-PLC inhibitors from the literature.

## 1. Introduction

**Figure 1:**
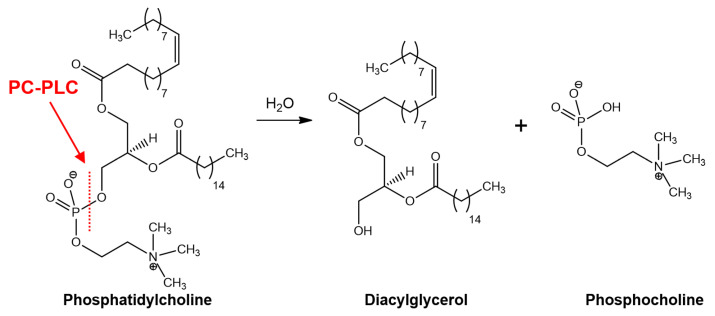
PC-PLC hydrolysis of phosphatidylcholine to diacylglycerol (DAG) and phosphocholine.

Phosphatidylcholine-specific phospholipase C (PC-PLC) is an enzyme that catalyzes the cleavage of phosphatidylcholine phospholipids to generate diacylglycerol (DAG) and phosphocholine as secondary messengers (Figure 1) [1]. DAG and phosphocholine mediate an array of cellular signaling proteins that maintain normal physiological functions in cells. DAG is well known for its role in the activation of protein kinase C (PKC), important for the activation of downstream proteins in several signal transduction cascades involved with, e.g., immune responses, cell growth and memory formation. The role of phosphocholine is not entirely clear, other than in synthesis of essential lipids in signal transduction [2]. Investigations into the pathophysiology of PC-PLC have revealed that the enzyme is pivotal to the development of several key pathological conditions. Therefore, PC-PLC has emerged as a potential biomedical target leading to the discovery of bespoke PC-PLC inhibitors. Details of the reported PC-PLC inhibitors and their pharmacological actions are outlined in this review.

**Figure 2:**
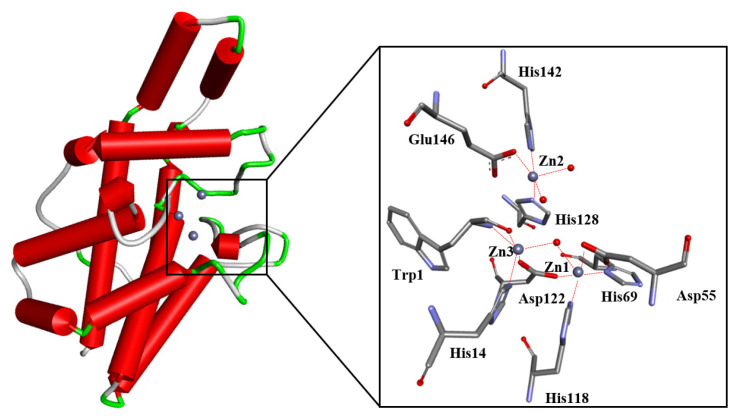
The structure of the wild-type PC-PLC*_Bc_* enzyme (PDB ID: 1AH7) and its catalytic site. The protein α-helixes are shown as red tubes. The trinuclear metal center consists of catalytic Zn^2+^ ions shown as grey spheres, whereas the water molecules are shown as red spheres. Amino acid residues coordinating to the Zn^2+^ ions are depicted and labelled. Figure edited from Eurtivong et al. [8].

Generally, studies into the biochemistry and pathophysiology of PC-PLC are relatively scant and less developed when compared to other phospholipase C subtypes such as phosphatidylinositol-specific phospholipase C (PI-PLC). Most notably, the genetic sequence of the mammalian PC-PLC has not yet been unambiguously identified. Recently, sphingomyelin synthases 1 and 2 were shown to exhibit PC-PLC-like activity [3], and are required for sphingomyelin homeostasis and growth in human HeLa cancer cells [4]. Thus, it is plausible that other enzymes could mimic PC-PLC activity (moonlighting function). Regardless, progress has been made towards understanding the mechanism of prokaryotic PC-PLC. A body of evidence suggests PC-PLC activity as a human functional equivalent of prokaryotic PC-PLC. The prokaryotic PC-PLC enzyme has been isolated and purified from *Bacillus cereus* (PC-PLC*_Bc_*), and its structure has been characterized (Figure 2) [5]. PC-PLC*_Bc_* was used as a model of its mammalian counterpart, e.g., they share antigenic similarities [6] and PC-PLC*_Bc_* is able to emulate similar responses in enhancing prostaglandin biosynthesis in mammalian cells [7]. The X-ray crystal structure is a monomeric zinc metalloproteinase with 245 amino acid residues [5]. The enzyme has thirteen α-helices with three Zn^2+^ ions in the active site. The Zn^2+^ ions are coordinated with water molecules and amino acid residues within the binding site; as shown in Figure 2, Zn1 is coordinated to a water molecule, Asp122, His69, Asp55 and His118; Zn2 is coordinated to two water molecules, His142, Glu146 and His128; and finally, Zn3 is coordinated to a water molecule along with Trp1 and Asp122.

## 2. Pathological Implications of PC-PLC

In this section, we summarize the implications of PC-PLC in pathophysiology based on what has been reported in the literature, specifically detailing the developments of pathological diseases and conditions, which include various types of cancers, atherosclerosis progression, induction of inflammation and neuronal cell death.

### 2.1. Cancer

The contributions of cancers to the global health burden are substantial: the World Health Organization has estimated approximately 10 million deaths in 2020 alone were caused by cancers; this figure is predicted to continue to grow [9]. In the past, significant progress has been made in cancer therapeutics. One of the major problems in cancer therapy is the continuous emergence of mutant proteins, which leads to significant mitigation of drug effectiveness. Thus, there is a strong drive towards the identification of alternative novel targets. One of these new anticancer targets that has gained some traction recently is PC-PLC. Some studies have identified PC-PLC activity to be involved in mediating intramolecular signals that lead to the induction of cancer-like properties. To little surprise, there have been studies suggesting choline metabolites and enzymes relevant to phospholipid homeostasis as biomarkers in monitoring tumor progression and response to therapeutic treatments, with some studies implying resistance to anticancer chemotherapy [10,11,12].

The liver is an organ with a major role in phospholipid metabolism that regulates turnovers of phospholipids via PC-PLC signaling [13]. Calcium-dependent activities of PC-PLC were reported to have been induced by a proliferative promoter, *N*-nitrosodiethylamine, in hepatocarcinogenic rats where PC-PLC activity peaked during tumor formation [14]. Likewise, using another proliferative promoter, phorbol 12-myristate 13-acetate, induced PC-PLC activity via PKC calcium-dependent pathway in CBRH-7919 rat hepatoma cells [14].

Breast cancer progressions are associated with aberrant signal transduction in cell proliferation. It is well-established that the PLC family, in particular PI-PLCs, mediates the signal transduction pathway by regulating several key membrane oncogenic receptor proteins, e.g., PLC-γ1 have roles in regulating EGFR activities [15], and PLC-δ4 is upregulated and enhances expression of HER2 and EGFR in MCF-7 breast cancer cells [16]. Similarly, a study showed that modulation of PC-PLC activities was able to regulate oncogenic HER2 and EGFR receptors: inhibition of PC-PLC reduced expression of HER2, induced HER2 internalization and reduced receptor recycling in SKBr3 cells and reduced membrane expression of EGFR and HER3 in SKBr3 cells, suggesting a strong relationship between PC-PLC signaling and its tumorous effects in breast cancer progression [17]. Additionally, in recent studies, the activation of PC-PLC has been implicated in the production of a significant portion (20–50%) of intracellular phosphocholine (PCho) within various subtypes of ovarian and breast cancer cells, suggesting PC-PLC as an active contributor to the progression of these cancers [18].

Ovarian cancer was responsible for ~300,000 deaths in 2020 [19]. The most common type of ovarian cancer is epithelial ovarian cancer (EOC), which is distinctively characterized by its ability to invade the abdominal cavity in the later stages. Resistance to platinum-based drugs is a common and unsolved problem. Recently, aberrant choline metabolism has been associated with EOC developments, with evidence of PC-PLC in sustaining the abnormalities of intracellular signaling in ovarian cancer cells [20]. Metabolic functions of PC-PLC were investigated in EOC cells, i.e., phosphocholine levels were detected to be 40–50% lower when PC-PLC was inhibited [18]. A similar protocol was used to show that elevated phosphocholine levels in HER2-overexpressed SKOV3 cells increase PC-PLC activity [21]. Additionally, PC-PLC activity was measured using the Amplex Red fluorescence assay in whole-cell lysates from a set of EOC cell lines with two-to-four-times higher activities in comparison to their non-tumoral counterparts [22].

Squamous cell carcinoma (SCC) is the second most prevalent form of skin cancer, accounting for approximately 23% of all skin cancers [23]. Tumor growth and proliferation of SCCs have been associated with PLC activities, e.g., PLC-γ was shown to be required for EGFR-induced mitogenesis and is overexpressed in human SCC cell lines [24], and PLC-γ has been implicated as a potential prognostic marker for oral SCC patients [25]. Specifically, PC-PLC activities were shown to drive oncogenic processes in human SCC cell lines, i.e., ×2.5 higher PC-PLC activities in an A431 human squamous cell line compared to non-tumoral keratinocytes were seen, and upon inhibition of PC-PLC, Western blot analysis revealed substantial decrease in EGFR phosphorylation activities [26].

Glioblastoma is an aggressive fast-forming type of brain cancer that originates in brain astrocytes. It represents the majority of brain cancers, and occurs in approximately 3 in 100,000 people [27]. Only 5% of patients are expected to live more than five years after diagnosis [27]. Existing treatments are considered ineffective, as the disease tends to recur at the same location [28]. Different forms of PLC have been associated with the developments of glioblastomas, including PLC-γ1 [29] and PLC-β1 [30]. CXCR4 is a G-protein coupled receptor that binds to chemokine ligands, which triggers a cascade in cancer-related signaling pathways that induce tumor growth and metastasis [31]. Neural stem cells have been noted to express high levels of CXCR4 receptors and can differentiate and develop into glioblastoma cells [32]. A recent report suggested that decreased expression of CXCR4 receptors following inhibition of PC-PLC in U87MG glioma cell models results in decreased proliferative and invasive activities, and suppresses EGFR and AKT kinase activities, suggesting modulation of PC-PLC can inhibit CXCR4 signaling and suppress glioblastoma growth [33].

### 2.2. Atherosclerosis

Atherosclerosis is a health condition resulting from the narrowing of the arteries due to accumulation of atherosclerotic plaque (atheroma) leading to increased risk of cardiovascular disease. It has been estimated that around 30% of deaths globally are associated with atherosclerotic–cardiovascular diseases [34].

A body of evidence suggests that PC-PLC signaling sustains the progression of atherosclerosis, suggesting PC-PLC as a potential drug target to treat cardiovascular diseases. It has been reported that PC-PLC signaling is induced by apolipoprotein C, a regulator of lipoprotein metabolism, which activates PKC and NF-κB, causing adhesion of monocytes to endothelial cells contributing to inflammatory responses in atherogenesis [35]. Inhibition of annexin A7, a phospholipid-binding protein, was shown to suppress PC-PLC activity in vascular endothelial cells and reduce atherosclerosis in apoE ^−/−^ mice [36]. Inhibition of PC-PLC in apoE ^−/−^ mice revealed suppression of LOX-1 receptor expression; LOX-1 is a key receptor of oxidized LDLs [37]. PC-PLC is a key mediator for NF-κB activation; in the study, it was also shown that NF-κB signaling is closely associated with expression of LOX-1 [37]. Reduced PC-PLC activities and apoptosis in vascular endothelial cells were reported during autophagy induced by sphingosylphosphorylcholine, a cardiovascular mediator with artheroprotective properties [38]. Additionally, it was reported that PC-PLC activities are mediated by PEBP1, a protein inhibitor of protein kinases, during atherosclerosis development, i.e., enhanced PC-PLC activities during elevated PEBP1 levels in atherosclerotic mice were reported, whereas PEBP1 downregulation was seen during PC-PLC inhibition [39].

### 2.3. Inflammation

Inflammation is a protective response of body tissues from harmful stimuli, and is regulated by a balanced combination of pro- and anti-inflammatory mediators [40]. However, an interference to this equilibrium can result in an excessive inflammatory response resulting in tissue damage [40].

The PLC family also mediate inflammatory responses given that DAG is a pro-inflammatory mediator. DAG activates PKC, which mediates various inflammatory responses in NF-κB and MAPK signal transduction pathways via extracellular signals including interleukins and TNFs [41,42,43]. Isotopic labeling of phosphatidylcholine indicated that hydrolysis via PC-PLC catalysis was enhanced by TNF stimulation, generating DAG mediators in monocytes and T cells [44]. Another report showed that IL-1 stimulation was able to enhance formations of DAG and phosphocholine products in T-lymphocytes in the absence of phosphatidylinositols, suggesting PC-PLC catalysis could be responsible for the generation of DAG secondary messengers via IL-1-mediated signal transduction [45].

### 2.4. Neuronal Cell Death

The central nervous system is responsible for the integration of sensory information and instructs body responses accordingly. Neuronal cell death is the essence of neurodegenerative diseases and injury, the second leading cause of mortality, responsible for an estimated nine million deaths per year [46].

There is some evidence indicating that suppressing PC-PLC activities may increase the growth and differentiation of neuronal cells, which suggests that PC-PLC could be a potential neurotherapeutic target. The glutamate/cystine antiporters are highly expressed in astrocytes that indirectly regulate intracellular levels of the antioxidant glutathione, through uptake of cystine in exchange for glutamate expulsion [47]. It was reported that high levels of glutamate are able to interfere with the antiporter mechanism via PC-PLC signaling that induces nerve cell death, possibly as a result of glutathione depletion, whereas inhibition of PC-PLC increases cell viability of the cortical cells [47]. Investigation into the effects of PC-PLC inhibition on vascular endothelial cells and marrow stromal cells revealed that the inhibition of PC-PLC signaling induced differentiation into neuron-like morphologies [48]; mechanistic studies have suggested that NADPH oxidation and an increase in ROS levels contribute to PC-PLC-mediated bone marrow stromal cell differentiation signal transduction pathways [49].

## 3. Discovery and Development of PC-PLC Inhibitors

In the past, a plethora of PI-PLC inhibitors have been discovered and developed, ranging from steroids [50,51], small molecules [52,53], natural products [54] and lipid analogues [55]. The lipid analogue edelfosine, a platelet-activating factor found in small concentrations in the human body that predominantly inhibit cytosolic fibroblast’s PLC-γ, was shown in clinical studies to treat various cancer types [55,56,57]. In contrast, there is a paucity of studies to develop PC-PLC inhibitors. Nevertheless, there have been recent discoveries of PC-PLC inhibitors that demonstrate glimpses of its therapeutic potential. In this section, details of different classes of the reported PC-PLC inhibitors are outlined. The most prominent PC-PLC inhibitors are given in Table 1.

### 3.1. 2-Aminohydroxamic Acids

The concept of using 2-aminohydroxamic acids as PC-PLC*_Bc_* inhibitors was mainly driven by Llebaria and coworkers [58,59]. These compounds were developed based on structural knowledge of PC-PLC*_Bc_* and aminopeptidases in *Streptomyces griseus* and *Aeromonas proteolytica* strains, e.g., triplet Zn^2+^ ion cofactors, as these enzymes may share similar active site architecture with human PC-PLC [59]. Derivatives of aminopeptidase inhibitors were designed and synthesized, including α-aminohydroxamic acids, and the most potent inhibitors are shown in Table 1. This was followed by experimental validation of PC-PLC*_Bc_* inhibition, with positive results. Pursuing from this success, modifications were introduced to the 2-aminohydroxamic scaffold to achieve stronger potencies, e.g., installation of choline linked to the hydroxamides with the prospect of stronger chelation to Zn^2+^ ions achieving IC_50_ values in the submicromolar range. Unexpectedly, the potencies tend to associate with amino acid binding rather than Zn^2+^ ion chelation. In the design of the series, enantioselectivity was not given priority, as it has been reported that the enzyme lacks enantiomeric selectivity.

### 3.2. Phospholipid Analogues

Substrate mimics have long been used in drug development, as they are able to imitate transition state conformations. This concept was implemented for the design and synthesis of phospholipid analogues as inhibitors of PC-PLC, and was pioneered by Martin and coworkers [60,61] (see Table 1). It was seen that modifications at the P-O phosphodiester was detrimental to activity. However, substitution of the glyceryl oxygen and oxygens at P=O and P-OH were tolerated. Modifications of side acyl chains favor six to eight carbon atoms, possibly mimicking the length of most PC-PLC*_Bc_* phospholipid substrates. Modifications at the acyl terminus seemed tolerable, with polar groups such as hydroxyl increasing solubility in aqueous environments.

### 3.3. Xanthates

PC-PLC inhibitors from the xanthate family primarily revolve around tricyclodecan-9-yl-xanthogenate (D609) with a *K_i_* value of 6.4 μM for PC-PLC*_Bc_* [62]. The chemical structure of D609 consists of a xanthate group linked to a merged tricyclic cyclodecane ring (see Table 1). The compound was initially discovered to have antiviral properties against DNA and RNA viruses [63]. Following this, D609 was discovered in other pharmacological applications, including having anticancer, anti-inflammatory, anti-atherosclerotic and anti-apoptotic properties. Interestingly, given D609’s pharmacological benefits, it received little interest in developing its structural activity relationship (SAR), most likely due to the non-drug likeness of the compound. Nonetheless, a study revealed that PC-PLC*_Bc_* lacked diasteromeric control towards the xanthates; eight pure D609 diastereomers were synthesized and evaluated in vitro with comparable potencies with IC_50_ values between 10 and 17 μM [64]. It was shown that the tricyclic system of D609 is not essential, i.e., replacement with *n*-decyl displayed a comparable *K_i_* value of 10 μM (see Table 1). Moreover, it was reported that shortening the length of alkyl chains and substituting with more bulky aromatic rings diminished PC-PLC*_Bc_* inhibition activities.

### 3.4. Pyrido[3,4-b]indoles

**Figure 3:**
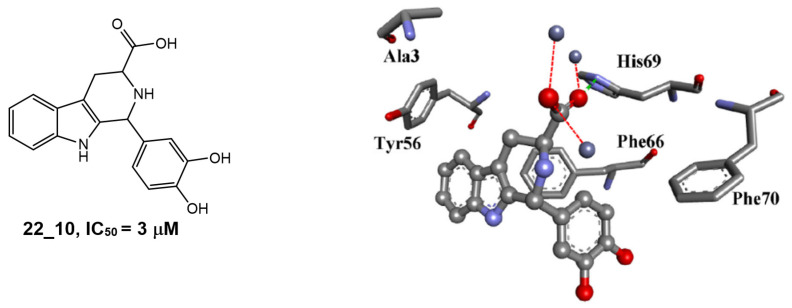
The chemical structure and potency of pyrido[3,4-*b*]indole derivative 22_10 as well as the predicted binding mode of (1*R*,3*S*)-22_10 derivative in the PC-PLC binding site. Figure edited from Eurtivong et al. [8].

The pyrido[2,3-*b*]indole is a prominent core scaffold in medicinal chemistry exhibiting a range of biological activities and mechanisms of action, e.g., anticancer [65], antileishmanial [66], NMDA antagonism [67], anti-depression [68] and estrogen antagonists [69]. Reynisson and coworkers [8] were the first to report the series as inhibitors of PC-PLC in a combined virtual screening SAR study. Molecular modelling studies revealed that the derivatives were able to chelate with the Zn^2+^ ions at the active site with the carboxylate functional group and interact with the imidazole ring in His69 (Figure 3). Only racemic mixtures of the pyrido[2,3-*b*]indoles were reported. Although molecular modelling suggests the (1*R*,3*S*)-diastereomer as the best inhibitor, there is as of yet no in vitro validation to confirm this finding. The chemical structure of a pyrido[3,4-*b*]indole derivative and the predicted binding interactions to the enzyme are shown in Figure 3.

### 3.5. Morpholinobenzoic Acids

The morpholinobenzoic acids were first reported to show PC-PLC*_Bc_* inhibition by Reynisson and coworkers [8]. A selection of inhibitors from the series are shown in Table 1. In that study, computational modelling was initially used to elucidate its binding mode to PC-PLC*_Bc_*, which demonstrated that the series chelates to the Zn^2+^ ions with the carboxylate functionality. Thus, it was initially assumed that the strong potencies observed were heavily reliant on this key interaction. However, good activities were maintained after derivatization of the carboxylic acid group to ester and hydroxamic acid, whilst complete removal of carboxylic acid improved activity for some derivatives [70,71,72]. In vitro tests for their antiproliferative effects revealed the carboxylate and ester derivatives displayed poor antiproliferative activities [71]. Nonetheless, the hydroxamic acid derivatives displayed promising antiproliferative activities against two cancer cell lines, e.g., 2-morpholinobenzene hydroxamic acid 9i (see structure in Table 1) had an IC_50_ value of 3.25 μM against MDA-MB-231 (epithelial human breast cancer), and IC_50_ = 5.8 μM against HCT-116 (human colorectal carcinoma) [71].

### 3.6. 1,4-Oxazepines

Recently, Zhao and coworkers [73] were the first to synthesize two chiral compounds, (*R*)-7-amino-2,3,4,5-tetrahydrobenzo[b][1,4]oxazepin-3-ol (*R*-7ABO) and (*S*)-7-amino-2,3,4,5-tetrahydrobenzo[*b*][1,4]oxazepin-3-ol (*S*-7ABO), based on the 1,4-oxazepine scaffold, and verified their PC-PLC*_Bc_* inhibition activities (see Table 1); the inhibition activities for the compounds were dose-dependent. Derivatives of 1,4-oxazepines have been implicated to an array of pharmacological activities, e.g., anti-convulsion [74], anti-diabetics [75], carbonic anhydrase inhibition [76], PI3K inhibition [77], and progesterone agonists [78].

### 3.7. N,N′-Dihydroxyureas

A selection of *N*,*N*′-dihydroxyureas were tested for PC-PLC activity due to successful coordination of polyhydroxy tropolones to bimetallic sites in enzymes. In a study conducted by Martin et al. [79], a bidentate ligand, 2,7-dihydroxytropolone (Table 1) was identified as a PC-PLC*_Bc_* inhibitor. Given this success, and knowing that hydroxamic acids are excellent Zn^2+^ chelators, derivatives of *N*,*N*′-dihydroxyureas were synthesized (as an example see derivative in Table 1). In the report, N-OH groups were incorporated into the rings for strong bimetallic Zn^2+^ chelation. Interestingly, PC-PLC inhibition experiments are pH-dependent, i.e., a high pH favors PC-PLC inhibition by *N*,*N*′-dihydroxyurea derivatives.

**Table 1:** Some PC-PLC inhibitors from literature.

Compound	Chemical Structure	IC_50_/*K_i_*	Assay	Reference
α-aminohydroxamic acid 6	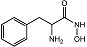	IC_50_ = 4 μM	Chromogenic-based assay	[59]
2-aminohydroxamic acid 18	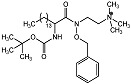	IC_50_ = 2 μM	Chromogenic-based assay	[58]
Phospholipid analogue 7	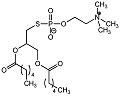	*K_i_* = 7 μM	pH-based assay	[60]
Dihydroxy phospholipid 7	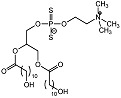	*K_i_* = 5.4 μM	Chromogenic-based assay	[61]
D609	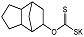	*K_i_* = 6.4 μM	Radiometric enzyme assay	[62]
Potassium *O*-decyl xanthate	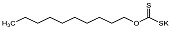	*K_i_* = 10 μM	Chromogenic-based assay	[64]
1*H*-pyrido[3,4-*b*]indole 22_10	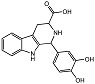	IC_50_ = 3.1 μM	Amplex Red assay	[8]
2-morpholinobenzoic acid 84	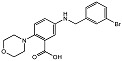	IC_50_ = 3.7 μM	Amplex Red assay	[8]
Morpholinobenzene 10k	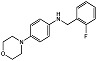	IC_50_ = 1.1 μM	Amplex Red assay	[70]
2-morpholinobenzene hydroxamic acid 9i	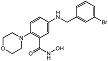	Unknown	Amplex Red assay	[71]
R-7ABO	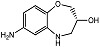	Unknown	Amplex Red assay	[73]
S-7ABO	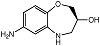	Unknown	Amplex Red assay	[73]
2,7-dihydroxytropolone	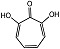	*K_i_*_(pH = 7.3)_ = 16 μM*K_i_*_(pH = 9.5)_ = 23 μM	Chromogenic-based assay	[79]
*N*,*N*′-dihydroxyurea 10	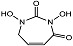	*K_i_*_(pH = 7.3)_ = 388 μM*K_i_*_(pH = 9.5)_ = 53 μM	Chromogenic-based assay	[79]

### 3.8. Univalent Anions

The PC-PLC*_Bc_* enzyme was reported to be inhibited by several univalent anions by coordinative interactions with the trinuclear Zn^2+^ ions. It was seen that univalent anions were most effective at inhibiting PC-PLC*_Bc_* activities as reported by Aakre and Little [80]. From the same study, phospholipid hydrolysis was substantially decreased in the presence of iodide, cyanate, chloride, nitrate and bicarbonate solutions. The univalent anions interact electrostatically with the trinuclear Zn^2+^ ions, which was confirmed by X-ray crystallographic data, e.g., complexation of iodide to Zn^2+^ ions in the PC-PLC*_Bc_* active site [81].

## 4. PC-PLC Enzymatic Assays

The ability to monitor enzyme activity accurately is a prerequisite for the development of enzyme inhibitors in modern medicinal chemistry. A reliable assay allows the efficacy of potential inhibitor molecules to be measured and quantified so that SAR can be established. In the last 30 years, a number of PC-PLC assays have been reported in the literature. Some of the earliest assays were low-throughput and/or required specialist reagents and instrumentations. For example, in 1994, Martin et al. conducted one of the first SAR studies on PC-PLC*_Bc_* by monitoring pH alterations during phosphatidylcholine hydrolysis [60]. Two years later, in 1996, Amtmann applied a radiometric assay that measured the rate of phosphorylcholine production by using ^3^H-labelled phosphatidylcholine substrates [62]. Although these methods yielded satisfactory results, their widespread application was hindered by their inherent limitations.

The first assay that was applied widely by the medicinal chemistry community was described by Hergenrother and Martin [82]. The assay allows PC-PLC activity to be measured in three indirect steps. The first one is enzyme-coupled, in which inorganic phosphate was released from the phosphomonoester product such as phosphocholine by the use of alkaline phosphatases. In the second step, the resulting inorganic phosphate ion is reacted with ammonium molybdate. The phosphate–ammonium molybdate complex is then reduced chemically using ascorbic acid in the last step. This results in a blue molybdenum chromogen, which can be observed through ultraviolet/visible (UV/vis) spectrophotometry with a maximum absorbance at 700 nm. As this assay can be conducted on a plate, it gained traction in the biochemical community and it has been applied in a number of inhibitor discovery studies for PC-PLC*_Bc_* [58,59,61,64,79]. However, as this assay is indirect and relies on both the activity of alkaline phosphatase and chemical reactions via ammonium molybdate and ascorbate, it is tedious and potentially prone to errors. In 2000, Flieger et al. developed a second UV/vis-based assay that allowed direct monitoring of PC-PLC activity [83]. By using α-naphthylphosphorylcholine (α-NPPC) as a (non-native) substrate, para-nitrophenol is generated as a product, which can be monitored spectrophotometrically with a maximum absorbance at 410 nm [83]. However, as this assay requires the use of non-native substrates, the results may not be directly translatable to the native system.

One of the most widely used assays to monitor PC-PLC activity is the so-called “Amplex Red assay” [84,85,86]. This assay utilizes a fluorogenic probe called 10-acetyl-3,7-dihydroxyphenoxazine (Amplex Red) to identify the presence of hydrogen peroxide. The hydrogen peroxide is generated through the conversion of phosphocholine (a product of PC-PLC-catalyzed reaction) to choline by alkaline phosphatase, which is followed by the oxidation of choline by choline oxidase. When hydrogen peroxide is present, it reacts with Amplex Red in the presence of horseradish peroxidase, resulting in the production of a highly fluorescent substance called resorufin. However, the Amplex Red assay has its own limitations, since the detection of PC-PLC activity is indirect and relies on multiple enzymatic and chemical conversion steps. For example, Sharma et al. found that the activity of horseradish peroxidase used in the assay may be inhibited by potential PC-PLC inhibitors, which may give false positive results [72].

Finally, several assays that allow direct detection of the turnover of native PC-PLC substrate have also been developed in the last decade. In 2017, Murakami et al. reported a liquid chromatography-mass spectrometry (LC-MS) assay to separate and quantify different PC-PLC substrates and products [87]. In 2021, Sharma et al. described a matrix-assisted laser desorption ionization time-of-flight (MALDI-TOF) mass spectrometry-based assay to monitor the formation of the diacylglycerol products [72]. In principle, these methods hold the potential for precise measurement and quantification of PC-PLC activity. However, as these assays were only published in the last few years, their novelty means that their uptake and adoption by the biochemical and medicinal chemistry communities are yet to be observed.

## 5. Conclusions and Future Perspective

PC-PLC has the potential to be a novel pharmacological target. It is apparent that PC-PLC is associated with developments of several pathological conditions and diseases, as outlined in this review. There is evidence at the molecular level for PC-PLC signaling being involved in the expression of oncogenic proteins. In addition, chemokine signaling has been linked to PC-PLC activity, which led to the progression of many cancers. As a consequence, this led to influences over various cellular processes such the regulation of cell cycles, signaling and proliferation. These observations are often supported by quantification of phosphocholine generation, and modulation of PC-PLC activities using small molecule PC-PLC inhibitors such as D609. Given DAG generation is a product of PC-PLC catalysis, it can be inferred that cancer is progressed via PKC signaling as the main route. There is evidence to suggest that PC-PLC is mediated by several signaling proteins and is involved in the development of atherosclerosis, albeit explanations to most of the underlying mechanisms remain unclear. Moreover, PC-PLC is suggested to activate inflammatory mediators via DAG and PKC signaling, and PC-PLC has been responsive to interleukin ligands. Lastly, reports of PC-PLC signaling associated with induction of nerve cell deaths via physiological mechanisms regulating glutathione levels, NADPH and ROS activities.

Despite some evidence indicating PC-PLC as a potential pharmacological drug target, it has so far received limited interest from the drug discovery community. Most of the findings were based on running biochemical assays to investigate PC-PLC activity and PC-PLC protein expression (detected by polyclonal antibodies against bacterial PC-PLC) in different cell-based models. Recent inhibitors that have been developed include drug-like inhibitors, indicating that PC-PLC has partly gained traction. Most of the inhibitors were conceptualized to chelate, or form electrostatic interactions, with the Zn^2+^ ions in the PC-PLC binding pocket as an important key feature. However, this concept was shown to be somewhat obscured, e.g., potencies of the 2-aminohydroxamic acids were reported with the tendency to correlate more with amino acid interactions rather than Zn^2+^ chelation. Furthermore, the removal of the carboxylic acid functionality from the 2-morpholinobenozic acid scaffold, or substitution with ester groups, were tolerated strongly, suggesting that the metal chelation hypothesis was invalid. Regardless, the PC-PLC*_Bc_* is commonly used as a model to study mammalian PC-PLC. The gene coding for the mammalian PC-PLC has not yet been identified, and as a result, the mammalian structure is unavailable. Given the lack of mammalian PC-PLC structural data, it remains questionable whether the compounds can actually inhibit the mammalian form. Therefore, the location of the corresponding human gene and characterization of the human PC-PLC structure is essential to verify the pharmacological applications of the current inhibitors, and future developments of PC-PLC inhibitors. Without the gene, the possibility remains that other enzymes serve as surrogates (moonlighters) in human physiology. The elucidation of the human PC-PLC gene is paramount in progressing the further development of PC-PLC as potential therapeutic target. Nevertheless, a body of biological data supports the case for the existence of PC-PLC in human physiology and several drug-like inhibitors have been proposed.

## Data Availability

Not applicable.
